# A Novel, Rapid, and Validated Stability-Indicating UPLC Method for the Estimation of Drotaverine Hydrochloride and Ibuprofen Impurities in Oral Solid Dosage Form

**DOI:** 10.3797/scipharm.1503-02

**Published:** 2015-04-13

**Authors:** Rekulapally Vijay Kumar, Vinay U. Rao, N. Anil Kumar, B. Venkata Subbaiah

**Affiliations:** 1 Department of Chemistry, J.N.T. University, Kukatpally, Hyderabad-500085, A.P, India; 2 Dr. Reddy’s Laboratories Ltd. IPDO, Bachupally, Hyderabad-500090, A.P, India

**Keywords:** Drotaverine, Ibuprofen, RP-HPLC, Stability-indicating, Impurities, ICH guidelines

## Abstract

A novel, stability-indicating, reversed-phase ultra-performance liquid chromatographic (RP-UPLC) method was developed for the determination of pure drotaverine hydrochloride and ibuprofen in the presence of their impurities and degradation products. The method was developed using a Waters UPLC BEH C18, 100 × 2.1 mm, 1.7 µm column with a flow rate of 0.3 mL/min and detector wavelength at 210 nm. The mobile phase consisted of potassium dihydrogen orthophosphate buffer and acetonitrile. Drotaverine hydrochloride and ibuprofen were subjected to the stress conditions of oxidative, acid, base, photolytic, and thermal degradation. Degradation products resulting from the stress studies were well-resolved, thus confirming the test method as stability-indicating. Validation of the method was carried out as per International Conference on Harmonization guidelines.

## Introduction

**Fig. 1:**
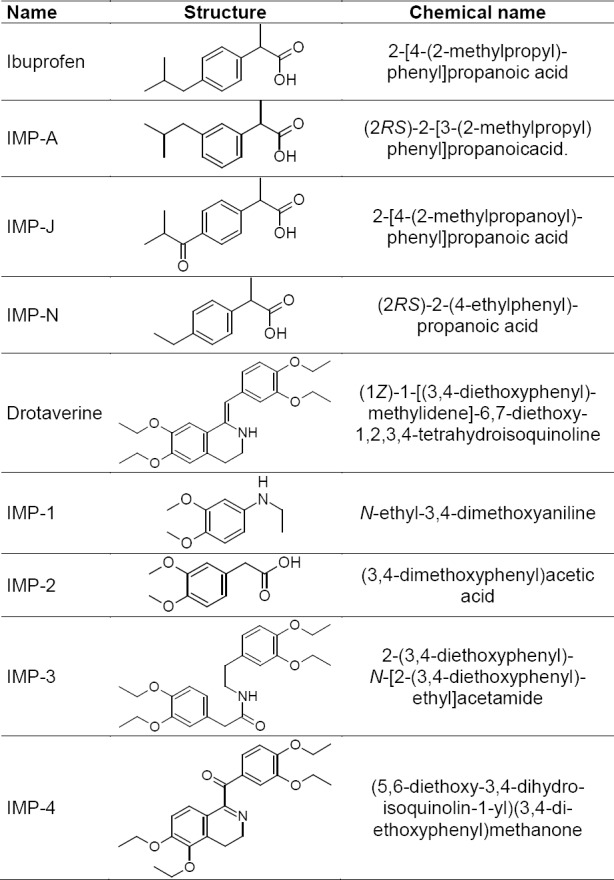
Chemical structure and name of ibuprofen, drotaverine, and impurities

Drotaverine hydrochloride is an antispasmodic. It is a benzylisoquinoline derivative described chemically as (1*Z*)-1-[(3,4-diethoxyphenyl)methylidene]-6,7-diethoxy-1,2,3,4-tetrahydroisoquinoline (Figure 1). Its empirical formula is C_24_H_31_ClNO_4_ and molecular weight is 433.5 g/mol [1–4].

Ibuprofen is a painkiller. It is chemically described as 2-[4-(2-methylpropyl)phenyl]propanoic acid (Figure 1). Its molecular formula is C_13_H_18_O_2_ and molecular weight is 206.29 g/mol [1–4].

A detailed literature survey revealed that there are some analytical methods reported for the estimation of drotaverine either individually or in combination with other drugs like HPTLC [5], spectrophotometric [6–8], and HPLC [8–13], and for ibuprofen either individually or in combination with other drugs like HPLC [14] and UPLC [15, 16]. The route of synthesis of drotaverine and possible degradants resulted in four known impurities: Impurity 1, Impurity 2, Impurity 3, and Impurity 4, which are not reported in any of the pharmacopeia, whereas ibuprofen EP revealed that Impurity A, Impurity F, Impurity J, and Impurity N are the known impurities for ibuprofen in which Impurity F detection was reported by gas chromatography.

To date, there is no single method that has been reported for the determination of drotaverine and ibuprofen combination impurities in either bulk drugs or in pharmaceutical formulations of drotaverine along with ibuprofen. It is necessary to develop a stability-indicating method for drotaverine and ibuprofen-related impurities in API and solid oral dosage forms which are available in combination packs.

Hence, an attempt has been made to develop an accurate, rapid, specific, and reproducible method for the determination of drotaverine and ibuprofen combination impurities (Figure 1) along with method validation as per ICH guidelines. The stability tests were also performed on drug products as per ICH guidelines [17–19].

There are no methods available for the simultaneous determination of drotaverine HCl and ibuprofen in any dosage forms. However, a number of methods are available for the quantification of related substances of plain ibuprofen and simultaneous estimation of ibuprofen (assay) with combination products. Two separate methods are available for drotaverine HCl and ibuprofen. To our present knowledge, no validated stability-indicating analytical HPLC or ultra-performance liquid chromatography (UPLC) methods are available in literature for drotaverine hydrochloride and ibuprofen and their impurities in oral dosage forms. Attempts were made to develop a stability-indicating UPLC method for the related substance determination of drotaverine hydrochloride and ibuprofen in the presence of a placebo. This paper deals with the forced degradation of drotaverine hydrochloride and ibuprofen oral solid dosage forms under stress conditions like acid hydrolysis, base hydrolysis, water hydrolysis, oxidation, and heat. This paper also deals with the validation of the developed method for the accurate quantification of drotaverine hydrochloride and ibuprofen impurities in oral solid dosage forms.

The method was developed on UPLC with low particle size (1.7 micron) and a short 100-mm column. A rapid, unique, reproducible, stability-indicating UPLC method was developed for the quantitative determination of drotaverine hydrochloride and ibuprofen impurities in oral solid dosage forms.

## Experimental

### Chemicals and Reagents

Drotaverine hydrochloride, ibuprofen standard, and their impurities were supplied by Dr. Reddy’s Laboratories (Hyderabad, India). The HPLC grade acetonitrile, analytical grade KH2PO4, and orthophosphoric acid were purchased from Merck (Darmstadt, Germany). High-purity water was prepared by using a Millipore Milli-Q Plus water purification system.

### Equipment

UPLC analysis was performed with a Waters (Milford, MA) Acquity UPLC system equipped with a quaternary solvent manager, sample manager, column-heating compartment, and photodiode array detector. This system was controlled by Waters Empower2 software.

An Acquity UPLC BEH C18 column, 100×2.1 mm, 1.7 µm (Waters) was employed for chromatographic separation. All samples were centrifuged by a Thermo Scientific Multifuge machine. The specificity study was conducted by using a heating oven (MACK Pharmatech, Hyderabad, India), photostability chamber, and waterbath equipped with a Millivolt controller (Julabo, Seelbach, Germany) which were used for the hydrolysis studies.

### Chromatographic Conditions

The method was developed using a BEH C18, 100×2.1 mm, 1.7 µm column as the stationary phase. Mobile phase A consisted of a mixture of 0.02 M phosphate buffer, pH 3.0, and acetonitrile in a 900:100 v/v ratio. Mobile phase B consisted of a mixture of a buffer, pH 3.0, and acetonitrile in a 400:600 v/v ratio. A mixture of Milli-Q water and acetonitrile in a 30:70 v/v ratio was used for diluent to prepare the solutions. The gradient program time (minutes)/% mobile phase B (%B) was set as 0/10, 6/50, 15/60, 18/65, 21/80, 23/100, 23.1/10, and 25/10, respectively. Before use, the mobile phase was mixed thoroughly and degassed. The mobile phase was pumped at 0.3 mL/min. The eluted compounds drotaverine hydrochloride, ibuprofen, and their impurities were monitored at 210 nm. The column temperature was maintained at 25°C. The injection volume for samples and standards was set at 1.0 µL.

### Preparation of Standard Solution and System Suitability Solution

Stock solutions of drotaverine 500 μg mL^−1^ and ibuprofen 1000 μg mL^−1^ were prepared by dissolving an appropriate amount in diluent individually. Working standard solution was prepared from the above stock solutions for related substances determination (2 μg mL^−1^ of drotaverine and 15 μg mL^−1^ of ibuprofen) in diluent.

System suitability solution was prepared by dissolving an appropriate amount of drotaverine and ibuprofen to get a concentration of 1000 μg mL^−1^ and 5000 μg mL^−1^, subsequently, in diluent and all impurities of drotaverine (2.0 μg mL^−1^) along with ibuprofen (15 μg mL^−1^) were mixed in the same preparation.

### Preparation of the Test Solution

Twenty individual tablets’ (drotaverine label claim: 80 mg per tablet, ibuprofen: 400 mg per tablet) contents were weighed and the average weight of each tablet was calculated. Tablet powder equivalent to 100 mg of the active pharmaceutical ingredient of drotaverine and 500 mg of ibuprofen was transferred into a 100-mL volumetric flask. To this, 70 mL of diluent was added and sonicated for 30 minutes with intermediate shaking. The solution was then diluted to 100 mL with diluent and centrifuged at 4000 rpm for 10 min. The supernatant was collected and used as sample solution; this solution was filtered through a 0.2-mm Millipore PVDF filter.

## Method Validation

The proposed method was validated as per ICH guidelines [33, 34].

### Method Validation Parameters

#### Specificity

Specificity is the ability of the method to measure the analyte response in the presence of its potential degradants. Stress studies were performed for the tablets to provide an indication of the stability-indicating property and specificity of the proposed method. Intentional degradation was attempted to determine the stress conditions of heat (60°C), photolytic sunlight for approximately 1.2 million lux hours and UV light, both at shorter and longer wavelengths for approximately 200 Wh/m^3^, acid (2 M HCl), base (2 M NaOH), and oxidation (3% H2O2) to evaluate the ability of the proposed method to separate drotaverine and ibuprofen from their degradation products. For the acid, base, water, hydrolysis, and oxidation conditions, the study period was 3 h in a water bath. For thermal degradation, it was stressed for 8 h. Humidity samples were exposed to 90% RH for 7 days. The peak purity test was carried out for the drotaverine and ibuprofen peaks by using a photodiode array (PDA) detector in the stress samples.

#### Precision

The precision of the related substances method was checked by injecting six individual preparations of drotaverine hydrochloride (1000 µg/mL), ibuprofen (5000 µg/mL), and their impurities with 0.20% of Imp-1, Imp-2, Imp-3, and Imp-4 for drotaverine, and Imp-A, Imp-J, and Imp-N for ibuprofen with respect to the analyte concentration. The relative standard deviation (RSD) of the area for each impurity was calculated.

Intermediate precision of the method was also evaluated using different analysts and different instruments in the same laboratory.

#### Limit of Detection (LOD) and Quantification (LOQ)

The limit of detection (LOD) and limit of quantification (LOQ) for the impurities and analytes (with respect to the unknown impurities) were determined at signal-to-noise ratios of 3:1 and 10:1, respectively, by injecting a series of dilute solutions with known concentrations. A precision study was also carried out at the LOQ level by injecting six individual preparations of ibuprofen and its known impurities (Imp-1, Imp-2, Imp-3, and Imp-4) and drotaverine hydrochloride and its known impurities (Imp-A, Imp-J, and Imp-N) and calculating the RSD of the area.

#### Linearity

Linearity test solutions for the related substance method were prepared by diluting stock solutions (described previously) to the required concentrations. The solutions were prepared at five concentration levels from the LOQ to 250% of the specification level, and 0.2% of the respective analyte concentrations of drotaverine hydrochloride (1000 µg/mL) and ibuprofen (5000 µg/mL). Correlation coefficient, value for the slope, Y-intercept, and percent bias of the calibration curve were calculated.

#### Accuracy

The accuracy study of all impurities was carried out in triplicate at the LOQ, 50%, 100%, 150%, and 250% of the target concentration level: 0.2% of the respective analyte concentrations of drotaverine hydrochloride (1000 µg/mL) and ibuprofen (5000 µg/mL). The percentages of recoveries for impurities were calculated.

#### Robustness

To determine the robustness of the developed method, experimental conditions were deliberately altered and the resolutions between all peaks were recorded. The flow rate of the mobile phase was 0.30 mL/min. To study the effect of flow rate on the resolution, flow was changed by 0.03 units from 0.27 to 0.33 mL/min. The effect of the column temperature on resolution was studied at 20 and 30°C instead of 25°C. The effect of the percent organic strength on resolution was studied by varying acetonitrile by +10% with a constant ratio of the buffer. Meanwhile, the other mobile phase components were held constant as stated previously.

## Results and Discussion

### Method Development and Optimization

The primary objective of the UPLC method was a reduction in run time to 25 min, without compromising the efficiency, compared with a run time of approximately 60 min on traditional LC analysis of the combined dosage form. The UPLC method reduces acetonitrile consumption (at least 80%) without compromising productivity and performance.

The method was optimized to separate the major degradation products formed under different stress conditions. The main target of the chromatographic method was to get the separation for closely eluting degradation products, mainly Impurity A and ibuprofen, which eluted very closely. At the same time, drotaverine, ibuprofen, and Impurity J also eluted closely. The degradation samples were analysed using different stationary phases like BEH C18, BEH C8, and mobile phases containing buffers like phosphate, acetate, and perchlorate with different pH’s (2.0–5.0) and using organic modifiers like acetonitrile and methanol in the mobile phase. The isocratic mode was not able to get the desired resolution between Impurity A and ibuprofen. The degradation products also were not separated with a proper resolution and also the degradation products of ibuprofen were not separating from the two actives. Hence, the method was optimized with a gradient program. As the concentration of drotaverine decreases when compared to ibuprofen to improve response, resolution, and peak shapes, the trials were executed with different columns and temperatures. Finally, the stated chromatographic conditions only resulted with proper separation, response, and acceptable peak shapes. It indicated that the gradient method with 10% acetonitrile as an organic modifier in mobile phase A and 60% in mobile phase B was successful in separating the drugs and all the chromatographic degradation products. Some of the trials are summarized as stated below.

### Selection of Stationary Phase:

**Fig. 2:**
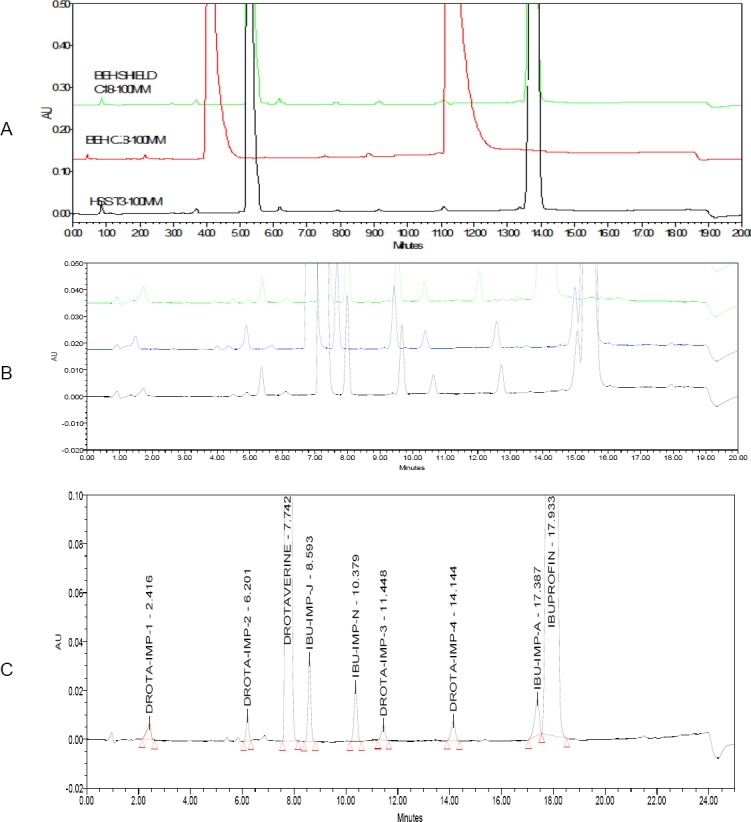
Method development chromatograms. (A) Selectivity differences of ibuprofen, drotaverine, and their impurities by using different stationary phases; (B) Retention of ibuprofen, drotaverine, and their impurities with gradient elution; (C) Blend chromatogram of ibuprofen, drotaverine, and their related impurities in final chromatographic conditions

The stationary phases like BEH C8, HSS T3, and phenyl columns were tried, but ibuprofen, drotaverine, and their impurities were not well-separated from the main peaks and the symmetry was not adequate as shown in Fig. 2A. On the other hand, the peak shapes for the two components were good and all the impurity peaks were well-separated from each other and their degradation products. Due to that, the column with BEH C18, 100×2.1 mm, and 1.7 µm stationary phase was selected.

### Selection of Mobile Phase Buffer:

The buffers like phosphate and acetate with pH’s of 4.5 and pH 7.5 and the perchlorate buffer with pH 2.5 and 3.2 were tried as a mobile phase buffer. However, with pH’s 4.5 and pH 7.5, as well as phosphate and perchlorate buffers with pH 2.5, the results were not good enough in terms of peak separation and peak shapes. On the other hand, the results with potassium dihydrogen phosphate buffer of pH 3.0 were found satisfactory so that the mobile phase with pH 3.0 phosphate buffer was finalized.

### Optimization of the Gradient Program:

Before going to the gradient program, an isocratic program with organic modifiers methanol and acetonitrile were tried, but the two components were eluted at the same retention time with both methanol and acetonitrile. Hence, the gradient method was used to separate all components from its known impurities. As the the column temperature was maintained at 30°C, acetonitrile was preferred as an organic modifier rather than methanol. The different gradient programs tried are shown in the Fig. 2B.

### Finalization of Chromatographic Conditions

By considering all the experiments, the chromatographic conditions were finalized as a BEH C18, 100×2.1 mm, 1.7 µm column with mobile phase containing a gradient mixture of solvents A and B, and a phosphate buffer (0.02 M) with pH 3.0. The buffer and acetonitrile in 900:100 v/v ratios were used as mobile phase A. The buffer and acetonitrile in 400:600 v/v ratios were used as mobile phase B. The gradient program time (minutes)/% mobile phase B (%B) was set as 0/10, 6/50, 15/60, 18/65, 21/80, 23/100, 23.1/10, and 25/10, respectively. The mobile phase was pumped at 0.3 mL/min. The eluted compound drotaverine hydrochloride, ibuprofen, and both of their impurities were monitored at 210 nm. The column temperature was maintained at 25°C. The injection volume for the samples and standards was 1.0 µL.

The chromatograph with finalized chromatographic conditions is shown in Fig. 2C.

### Validation of the Method

#### System Suitability

System suitability parameters were measured to verify the system, method, and column performance. Results of other system suitability parameters such as relative retention time of each impurity, tailing factor, and similarity factor (between two preparations) are presented in Table 1. As the data show, the acceptable system suitability parameters are: relative retention time of each impurity should be comparable, tailing factor for drotaverine hydrochloride and ibuprofen in standard solution should not be more than 2.0, and similarity factor (between two standard preparations) should not be less than 0.98 and not more than 1.02. Resolution between all peaks should be more than 1.5.

**Tab. 1:**
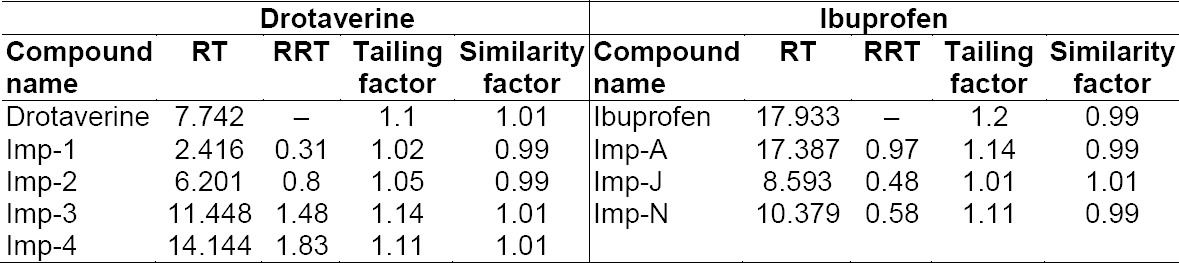
System suitability parameters

#### Specificity

**Fig. 3:**
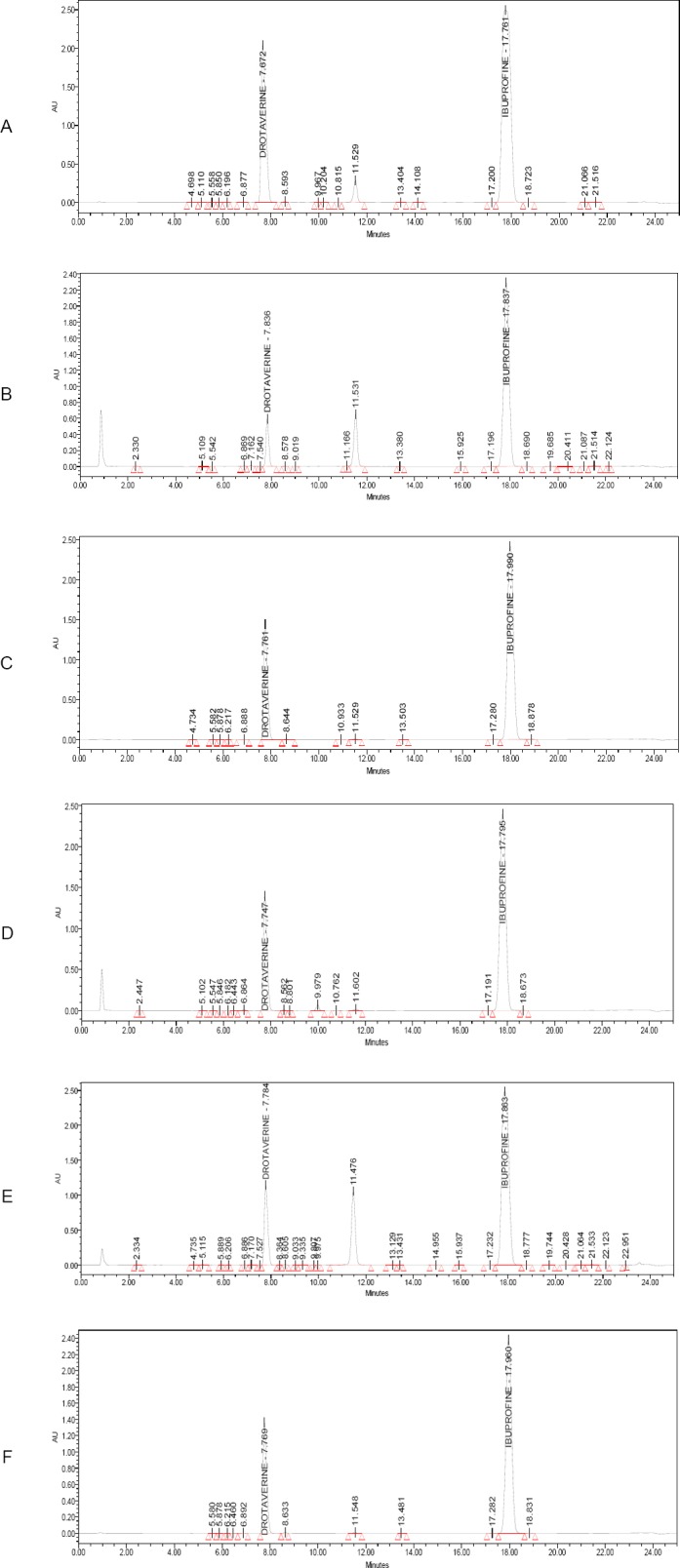
Typical chromatograms of ibuprofen and drotaverine under stress conditions: (A) acid hydrolysis, (B) base hydrolysis, (C) water hydrolysis, (D) peroxide degradation, (E) thermal degradation, (F) photodegradation

All forced degradation samples were analyzed at an initial concentration of drotaverine hydrochloride and ibuprofen with previously described UPLC conditions using a PDA detector to ensure the homogeneity and purity of the drotaverine and ibuprofen peaks. Significant degradation of drotaverine and ibuprofen was observed in heat (60°C for 3 h), photolytic UV light (200 Wh/m^3^), sunlight (1.2 million lux hours), oxidation (3.0% H_2_O_2_ at 24 h RT), acid (2 M HCl at 60°C for 3 h), base (2 M NaOH at 60°C for 3 h), and humidity exposed to 90% RH for 7 days. The percent degradation values are presented in Table 2 and Figures 3A-F.

**Tab. 2:**
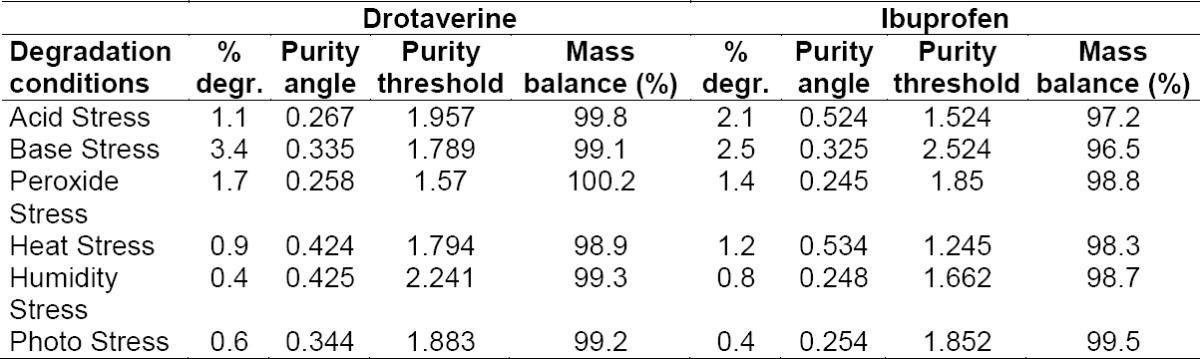
Summary of results from forced degradation studies

#### Precision

The RSDs for the areas of drotaverine Imp-1, Imp-2, Imp-3, and Imp-4 and ibuprofen Imp-A, Imp-J, and Imp-N in related substances are presented in Table 3. The method precision and intermediate precision study were found to be less than 2% as shown in Table 4 (which should be less than 10.0%), confirming good precision of the method.

**Tab. 3:**
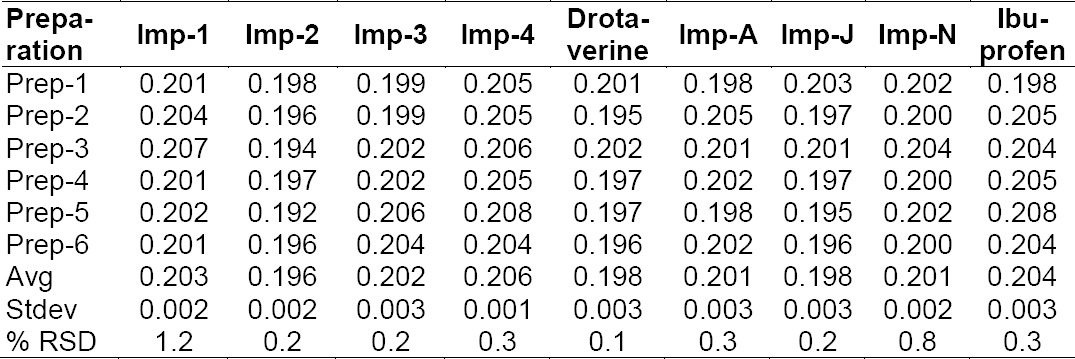
Precision of the proposed HPLC method

#### LOD and LOQ

The determination of the LOD and LOQ of drotaverine hydrochloride and its impurities Imp-1, Imp-2, Imp-3, Imp-4, and ibuprofen and its impurities Imp-A, Imp-J, Imp-N are established in this method. The precision values at the LOQ concentrations for drotaverine hydrochloride along with its impurities Imp-1, Imp-2, Imp-3, and Imp-4 and ibuprofen along with its impurities Imp-A, Imp-J, and Imp-N were found to be below 5% (which should be less than 10.0%). The LOD and LOQ of all the impurities’ values are presented in Table 5.

**Tab. 5:**
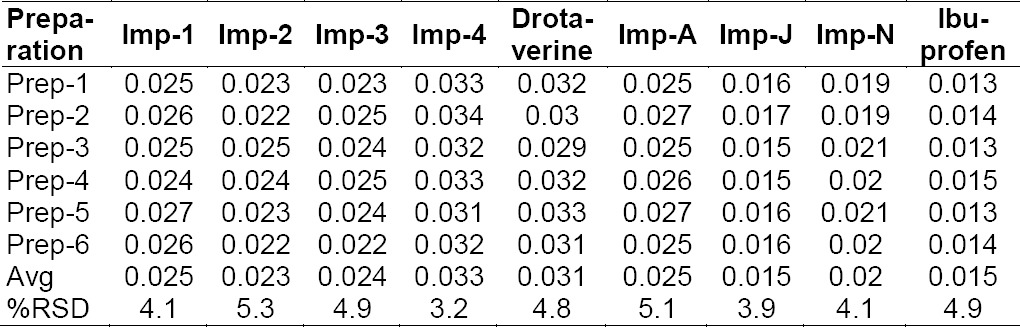
Precision at the LOQ

#### Linearity

The results show that an excellent correlation existed between the peak area and concentration of the analyte. A linear calibration plot for the related substances method was obtained over the calibration ranges tested, i.e., the LOQ to 250% for drotaverine and its impurities (Imp-1, Imp-2, Imp-3, and Imp-4) and ibuprofen and its impurities (Imp-A, Imp-J, and Imp-N). The correlation coefficient obtained was greater than 0.997 (Table 6). These results show that an excellent correlation existed between the peak area and the concentration. The percent bias was also calculated for all related compounds and main analytes and was found to be less than 2.5% (Table 6).

**Tab. 6:**
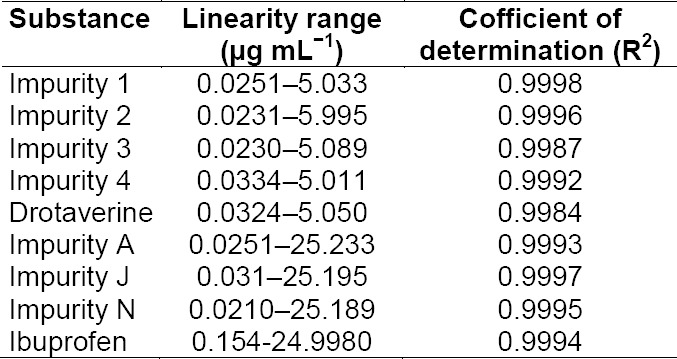
Linearity of the proposed HPLC method

#### Accuracy

The percentage recovery of drotaverine hydrochloride and ibuprofen impurities varied from 85 to 115% and the RSD of the three samples at each level was found to be less than 15% at 50%, 100%, 150%, and 250% levels of the target 0.2% level of the target concentrations, respectively. An LC chromatogram of the spiked sample at the 0.2% level of all seven impurities and two main peaks in the oral solid dosage sample solution is shown in Figure 2. Percent recovery values for the impurities are presented in Table 7 (percent recovery should be between 90 and 110%).

**Tab. 7:**
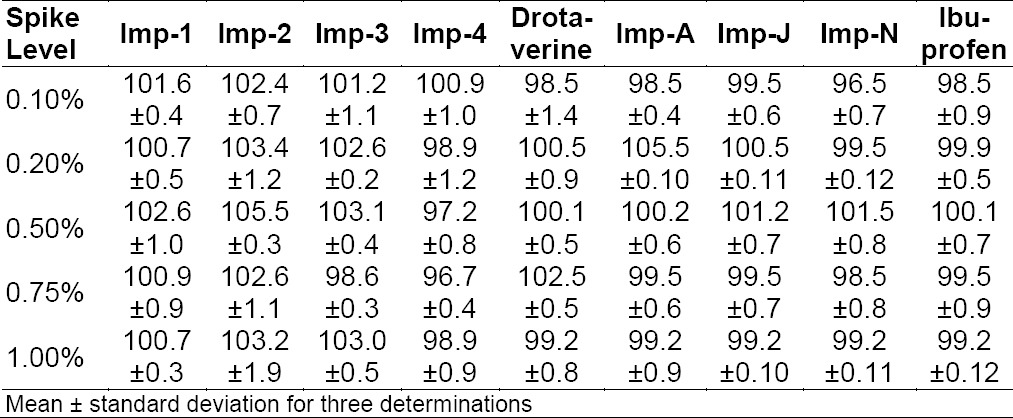
Accuracy of the proposed HPLC method

#### Robustness

In all of the deliberately varied chromatographic conditions (flow rate, column temperature, and composition of organic solvent), the resolution between the critical pairs was greater than 2.0, illustrating the robustness of the method.

## Conclusion

The gradient UPLC method which was developed for drotaverine hydrochloride, ibuprofen, and their related substances in oral solid pharmaceutical dosage forms was found to be precise, accurate, linear, robust, rugged, and specific. Satisfactory results were obtained from the validation of the method. Hence, the method is stability-indicating and can be used for routine analysis of production samples and to check the stability samples of oral solid dosage forms.
